# Sleep-Disordered Breathing in Chung–Jansen Syndrome

**DOI:** 10.3390/ijms27041736

**Published:** 2026-02-11

**Authors:** Katerina Vlami, Konstantina Kosma, Lamprini Athanasopoulou, Eirini Kokiou, Vasileios Paraschou, Maria Gerogianni, Stylianos Loukides, Melpomeni Peppa

**Affiliations:** 1 2nd Pulmonary Medicine Department, Medical School, General University Hospital “Attikon”, National and Kapodistrian University of Athens, Rimini 1 Str., 12462 Haidari, Greece; athanasopouloulamp@gmail.com (L.A.); ekokiou@yahoo.gr (E.K.); billisparas@gmail.com (V.P.); loukstel@med.uoa.gr (S.L.); 2 Laboratory of Medical Genetics, St. Sophia’s Children’s Hospital, National and Kapodistrian University of Athens, 11527 Athens, Greece; kkosma50@gmail.com; 3 Endocrine Unit, 2nd Department of Propaedeutic Internal Medicine, Attikon University Hospital, School of Medicine, National and Kapodistrian University of Athens, Rimini 1 Str., 12462 Haidari, Greece; gerogianni.e.maria@gmail.com (M.G.); moly6592@yahoo.com (M.P.); 4 3rd Department of Internal Medicine, Sotiria General Hospital for Chest Diseases, Medical School, National and Kapodistrian University of Athens, 11527 Athens, Greece

**Keywords:** pleckstrin homology domain interacting protein, genetic obesity, obstructive sleep apnea

## Abstract

We report a thirty-six-year-old woman with intellectual disability who was referred for evaluation of suspected obstructive sleep apnea. The initial clinical impression suggested a syndromic case, so comprehensive genetic testing was undertaken. Overnight polysomnography revealed a severe rapid eye movement–predominant obstructive sleep apnea syndrome with an apnea–hypopnea index of 31.9 events per hour, rapid eye movement apnea–hypopnea index of 113.8 events per hour, and lowest oxygen saturation of 66%. Treatment with continuous positive airway pressure improved respiratory and sleep quality indices and was well tolerated. Whole-exome sequencing identified a de novo splice site variant in the pleckstrin homology domain interacting protein gene (c.41-1G > A), confirming a molecular diagnosis of Chung–Jansen Syndrome. Chung–Jansen syndrome is a rare neurodevelopmental disorder caused by heterozygous pathogenic variants in the pleckstrin homology domain interacting protein gene, marked by developmental delay, intellectual disability, behavioral abnormalities, dysmorphism, and progressive obesity. PHIP influences central and peripheral pathways controlling satiety, pancreatic function, and body weight. Despite frequent reports of sleep problems, systematic evaluation of sleep-disordered breathing has been limited. This adult case provides the first polysomnographic confirmation in the syndrome, supporting proactive screening for obstructive sleep apnea—especially in those with obesity. Integrating genetic assessment into sleep care can reduce diagnostic delays and better guide therapy and prognosis.

## 1. Introduction

Chung–Jansen syndrome (CHUJANS; Online Mendelian Inheritance in Man database #617991) is a very rare genetic disorder first delineated in 2018 [1]. Pathogenic variants in the pleckstrin homology domain-interacting protein (PHIP) gene cause a core phenotype of developmental delay and/or intellectual disability (DD/ID), behavioral disturbance, hypotonia, progressive obesity, and a recognizable craniofacial gestalt (e.g., prominent eyebrows, large ears or earlobes, anteverted nares, and a long philtrum) [2]. Variants are usually de novo, though autosomal-dominant inheritance occurs; the spectrum includes nonsense, frameshift, missense, and deletions, with loss of function leading to haploinsufficiency or reduced protein function [3]. PHIP1 contains tryptophan–aspartic acid (WD40) repeats, a pleckstrin homology (PH)-binding region, two bromodomains, and a nuclear localization signal [3]. It also functions as a Damage-Specific DNA-Binding protein 1 (DDB1)- and Cullin-4 Scaffold protein (CUL4)-associated factor (DCAF14) substrate receptor within the (CUL4)–(DDB1)-Cullin–RING E3 ubiquitin ligase (CRL4) complex, which regulates cell proliferation, survival, and DNA repair [4,5]. Beyond neurodevelopment, PHIP integrates metabolic control by binding insulin receptor substrate-1 (IRS-1), modulating insulin receptor signaling, supporting pancreatic beta-cell growth and survival, and, through its nuclear isoform, promoting the transcription of proopiomelanocortin (POMC), a key satiety neuropeptide [6,7,8,9]. Disruption of these peripheral and central pathways offers a mechanistic basis for dysregulated appetite, altered energy balance, insulin resistance, and the high prevalence of obesity in affected individuals; weight often worsens with age, although some children initially present with normal weight. The main differential diagnosis is Prader–Willi syndrome (PWS), but facial gestalt and confirmatory genetics distinguish CHUJANS [10].

Sleep difficulties are noted in clinical series with CHUJANS, and adenotonsillar surgery appears relatively common, suggesting upper-airway vulnerability; however, no published cohort has systematically evaluated obstructive sleep apnea (OSA) using polysomnography (PSG) (see Supplementary Table S1). This report documents polysomnographically confirmed OSA in an adult with genetically confirmed CHUJANS, emphasizing the clinical value of systematic sleep evaluation in neurodevelopmental disorders characterized by obesity and intellectual disability, without implying a causal relationship between Chung–Jansen syndrome and OSA. We therefore describe the first adult case of CHUJANS with severe rapid eye movement (REM) predominant OSA confirmed by PSG, accompanied by a focused report extracting demographics, weight status, and sleep-related symptoms, surgeries, or sleep study findings from published PHIP/CHUJANS cohorts.

## 2. Detailed Case Description

A 36-year-old woman was referred to our Sleep Unit for evaluation of snoring, witnessed apneas, and excessive daytime sleepiness. Her medical history was notable for morbid obesity (body mass index [BMI]: 48 kg/m^2^), intellectual disability, generalized hypotonia, epilepsy (well controlled on stable antiepileptic therapy), autoimmune hypothyroidism (Hashimoto disease, adequately treated with levothyroxine), and hyperlipidemia. Clinical examination revealed characteristic dysmorphic features, including large earlobes and prominent eyebrows. The oropharyngeal airway was classified as Mallampati class IV. Her neck circumference was 44 cm and waist circumference was 139 cm. Excessive daytime sleepiness was confirmed using the Epworth Sleepiness Scale (ESS), with a score of 12 (normal < 10). A full-night attended PSG with synchronized audio–video recording was conducted, which remains the gold standard for the diagnosis of sleep-disordered breathing. PSG records multiple physiological signals during sleep, including electroencephalography (EEG), electrooculography (EOG), electromyography (EMG), airflow, thoracoabdominal respiratory effort, oxygen saturation, heart rate, body position, and snoring.

**Figure 1:**
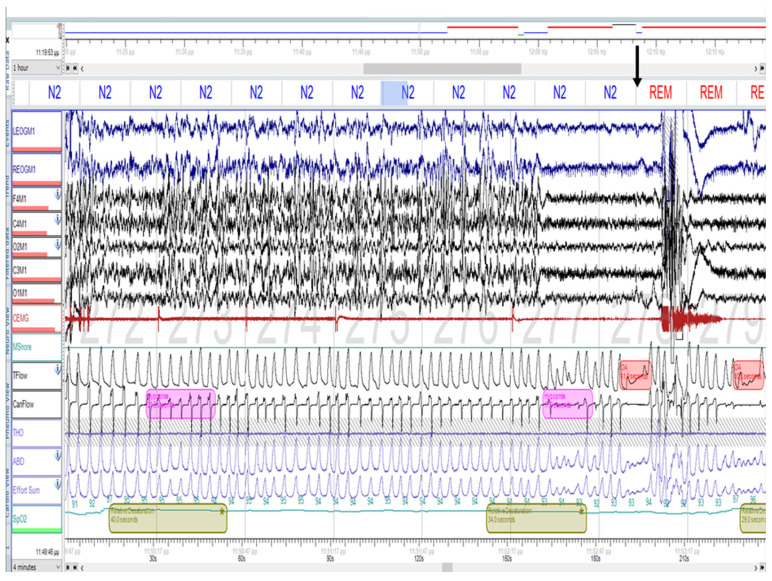
Representative attended overnight polysomnography from a 36-year-old woman with Chung–Jansen syndrome, showing a 4 min recording segment (consecutive eight 30 s epochs) of electroencephalography (F4M1, C4M1, O2M1, C3M1, O1M1), electrooculography (LEOGM1, REOGM1), chin electromyography (CEMG), airflow (Tflow, Canflow), thoracoabdominal effort (THO, ABD, Effort Sum), and oxygen relative desaturation (SpO_2_, light green bars). Obstructive apneas (red bars) and hypopneas (pink bars) are illustrated during the transition from sleep stage N2 to REM sleep (black arrow).

The study was performed using standard procedures and equipment in accordance with the American Academy of Sleep Medicine (AASM) guidelines for performing polysomnography. PSG data were scored manually by a certified sleep technologist (EK) according to the AASM 2012 scoring criteria [11], and all recordings were subsequently reviewed by a sleep medicine specialist (KV). Obstructive apnea was defined as a ≥90% reduction in airflow lasting ≥10 s. Hypopnea was defined as a ≥30% reduction in airflow lasting ≥10 s and associated with either a ≥3% oxygen desaturation or an arousal. The apnea–hypopnea index (AHI) was calculated as the total number of apneas and hypopneas per hour of sleep. OSA severity was classified according to the AHI as mild (5.0–14.9 events/hour), moderate (15.0–29.9 events/hour), or severe (≥30 events/hour) (Figure 1).

**Figure 2:**
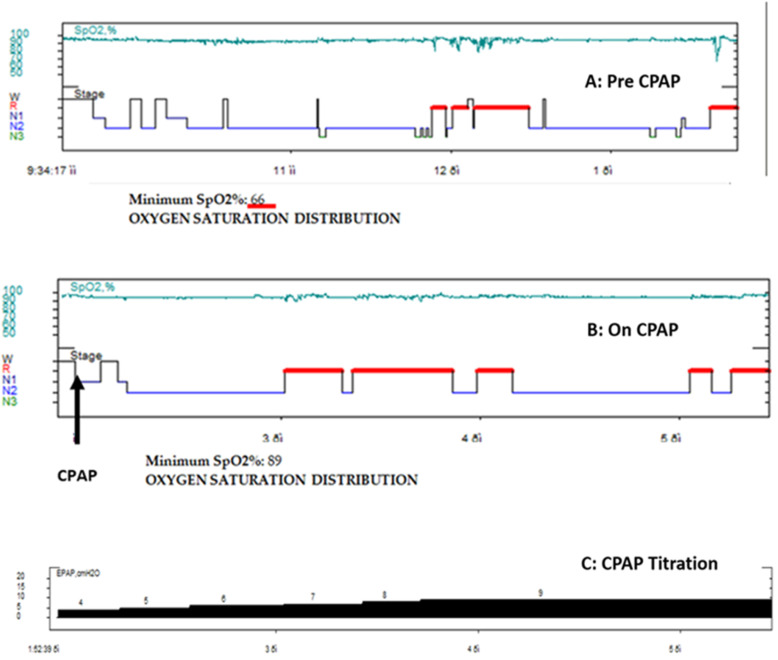
Oxygen saturation and sleep architecture during split-night polysomnography. Panel A depicts the diagnostic portion of the study prior to continuous positive airway pressure (CPAP) initiation, showing oxygen desaturations with a minimum SpO_2_ of 66% (green line). Panel B illustrates the CPAP titration portion; CPAP initiation is indicated by a black arrow, followed by stabilization of oxygen saturation (minimum SpO_2_ 89%) (green line). Prolonged, consolidated REM periods (horizontal red lines) are observed. Panel C depicts the progressively increased pressure of the CPAP with effective elimination of obstructive respiratory events at approximately 7–8 cm H_2_O.

A split-night protocol was followed. After confirming OSA in the first half of the night, continuous positive airway pressure (CPAP) was administered and titrated manually to optimize control of respiratory events. Respiratory indices normalized with improvement in oxygenation and evidence of REM rebound due to the chronic disturbed sleep architecture (Figure 2). In this case, the patient demonstrated severe OSA, with an AHI of 31.9 events/hour, characterized by REM sleep-predominant obstructive respiratory events (Table 1).

During the titration portion of the split-night study, obstructive respiratory events were effectively eliminated at CPAPs of approximately 8 cm H_2_O, with stabilization of oxygen saturation at 9 cm H_2_O; based on these findings, an auto-adjusting CPAP device was prescribed (pressure range: 5–9 cm H_2_O) (Figure 2).

**Figure 3:**
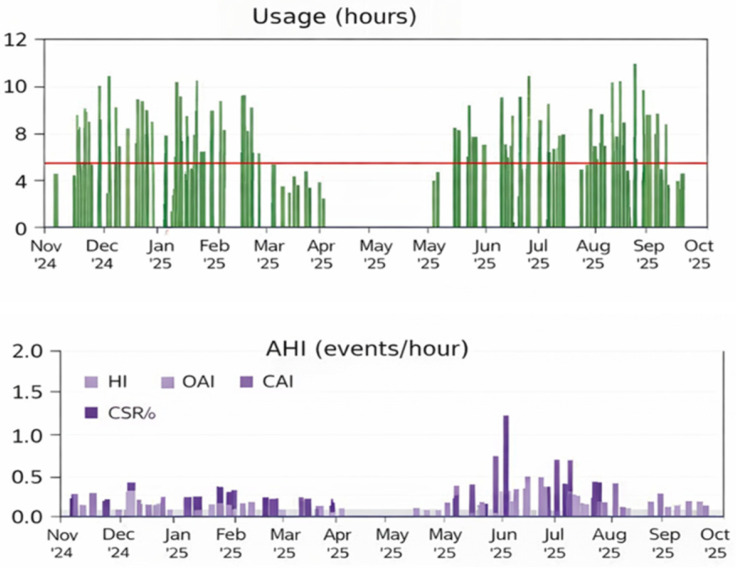
Summary of continuous positive airway pressure (CPAP) therapy. The upper panel shows nightly CPAP usage over the follow-up period (green lines). The lower panel illustrates the apnea–hypopnea index (AHI) events per hour of sleep over the follow-up period (purple lines).

At the 10-month follow-up, CPAP adherence remained satisfactory, and the patient reported notable symptomatic improvement regarding daytime sleepiness and snoring (Figure 3). A glucagon-like peptide-1(GLP-1) receptor agonist was also trialed for weight management but was discontinued due to adverse neuropsychiatric effects.

Finally, whole-exome sequencing identified a de novo splice-site variant in PHIP (c.41-1G > A), classified as pathogenic according to the American College of Medical Genetics and Genomics variant classification framework: Pathogenic Strong 2 (PS2), Pathogenic Very Strong (PVS1), Pathogenic Moderate 2 (PM2). Parental testing confirmed the variant was de novo.

## 3. Discussion

Here, we describe the first documented adult case of severe OSA in CHUJANS, extending the literature, in which prior series reported only sleep complaints but provided no PSG-confirmed sleep data. Our case shows that clinically significant REM-predominant OSA can occur in CHUJANS and responds well to CPAP. This observation likely reflects the intersection of central obesity, generalized hypotonia, and craniofacial morphology, which together increase upper-airway collapsibility—especially in REM sleep, when tonic activity of pharyngeal dilator muscles is at its lowest [12].

**Figure 4:**
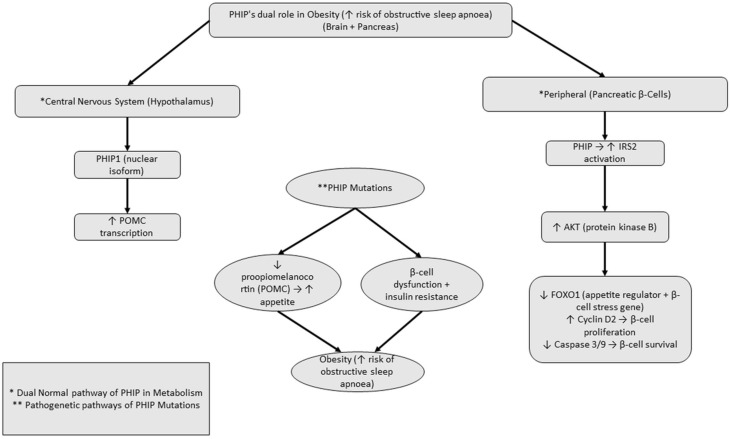
PHIP in central and peripheral pathways regulating appetite and metabolism. In Chung–Jansen syndrome, PHIP in central and peripheral pathways regulate appetite and metabolism. In the hypothalamus, pleckstrin homology domain interacting protein 1 enhances insulin/insulin-like growth factor 1 signaling via insulin receptor substrate 2, promoting AKT activation, the inhibition of forkhead box O1, and increased proopiomelanocortin transcription (satiety). In pancreatic β-cells, the same PHIP–IRS2–AKT axis supports β-cell proliferation, survival, and insulin secretion. Pathogenic variants in PHIP disrupt these pathways, contributing to hyperphagia, insulin resistance, and obesity, which may increase vulnerability to obstructive sleep apnea. In the figure (*) denotes pathways involving both central and peripheral mechanisms, whereas double asterisks (**) indicate pathogenic PHIP gene mutations. *Abbreviations: PHIP, pleckstrin homology domain-interacting protein; PHIP1, nuclear isoform; IRS2, insulin receptor substrate 2; AKT, protein kinase B; FOXO1, forkhead box O1; POMC, proopiomelanocortin; IGF-1, insulin-like growth factor 1; OSA, obstructive sleep apnea*.

CHUJANS, is a very rare genetic syndrome, which, beyond the neurodevelopmental and craniofacial features, is commonly characterized by early-onset, progressive obesity, which is likely mechanistically linked to PHIP dysfunction. The nuclear isoform PHIP1 normally augments insulin growth factor 1 (IGF-1) signaling via IRS2, enhancing Protein Kinase B (AKT) activation, supporting pancreatic β-cell proliferation, survival, and insulin secretion. In the hypothalamus, AKT phosphorylates and inhibits a transcription factor, the forkhead box O1 (FOXO1), relieving its repression of POMC and thereby promoting satiety. Pathogenic PHIP variants disrupt this axis, diminishing AKT-mediated FOXO1 inhibition, reducing POMC expression (predisposing to hyperphagia), and impairing β-cell function, thereby predisposing patients to insulin resistance (Figure 4).

The dual central–peripheral impairment of PHIP dysfunction plausibly underlies the characteristic childhood-onset weight gain in CHUJANS. Given the established association between obesity and OSA and the age-related rise in overweight status in CHUJANS, the cumulative risk for OSA is expected to increase over time in individuals with this rare syndrome. Although the current literature shows that overweight and obesity in CHUJANS are common and increase with age, the diagnosis of sleep-disordered breathing—particularly in the context of obesity—remains notably underexplored, despite increasingly robust descriptions of cognitive, and behavioral features.

In an early cohort studied by Jansen et al. [1], sleep problems were mentioned only anecdotally in clinical summaries, without systematic assessment (e.g., PSG, actigraphy) or subtyping (e.g., insomnia, circadian rhythm disorders, sleep-disordered breathing).The Craddock et al. [3] cohort (*n* = 10) likewise examined genotype–phenotype variability in detail but did not evaluate sleep quality, breathing disturbances, or responses to treatments such as CPAP or adenotonsillectomy; isolated upper-airway surgical interventions were noted but not linked to objective sleep outcomes. More recent series highlight that caregiver-reported sleep difficulties are common yet still poorly characterized: reported prevalence rises from ~18% [1] to 26.1% [6] and 42.6% [2], with the latter likely reflecting structured probe questions during interviews rather than a true epidemiologic shift. Even so, these studies did not subtype sleep disorders or report PSG data (see Supplementary Table S1).

Notably, upper-airway pathology appears frequent in CHUJANS (e.g., adenotonsillectomy/tonsillectomy in ~31.9% of reported cases). Combined with the syndrome’s hypotonia and age-related weight gain, these features plausibly elevate the risk of OSA; nevertheless, key sleep metrics—OSA prevalence, REM- or supine-predominant patterns, and hypoxic burden—remain unreported in current cohorts.

Positive airway pressure (PAP) therapy for OSA—though often challenging in neurodevelopmental disorders—should be considered as a trial option, as it may improve sleep and, secondarily, cognitive functioning. In our case, CPAP was well tolerated, alleviating nocturnal apneas and improving daytime sleepiness. This contrasts with experience in PWS, where CPAP adherence is often suboptimal and sustained use can be difficult [13]. The better tolerance observed in our patient highlights the potential benefit of PAP therapy in CHUJANS and supports its inclusion in routine clinical management.

Another noteworthy observation in our case was the adverse reaction to anti-obesity treatment with a GLP-1 receptor agonist, which had to be discontinued due to the emergence of severe neurological symptoms. While the growing use of anti-obesity medications—particularly GLP-1 receptor agonists—in the management of OSA reflects an evolving understanding of the pathophysiological link between obesity and sleep-disordered breathing [14], caution is warranted in patients with syndromic or genetically driven obesity. In CHUJANS, for example, pathogenic PHIP variants disrupt both central (POMC/FOXO1) and peripheral (IRS2/AKT) metabolic signaling pathways, resulting in a complex neuroendocrine profile that may alter drug responses. As demonstrated in our patient, pharmacologic interventions targeting metabolic pathways may lead to unpredictable or even paradoxical outcomes.

Additionally, this case suggests that in young adults with obesity and cognitive or behavioral difficulties, clinicians should remain cautious for underlying genetic syndromes. Recognizing and treating OSA in this context is critical, as untreated OSA may itself exacerbate cognitive impairment and obscure the underlying diagnosis.

Furthermore, understanding the genetic basis of obesity—such as PHIP-related mechanisms in CHUJANS—could open new avenues for gene-informed or gene-targeted therapies tailored to each patient’s molecular profile, addressing not only obesity itself but also its associated conditions, including OSA [9].

The limitation of this report is that as a single case, causality cannot be inferred; nonetheless, it highlights a testable hypothesis that OSA is underrecognized in CHUJANS. Prospective cohorts with systematic PSG are needed. Mechanistic work linking PHIP pathways (e.g., POMC regulation) to ventilator control and adiposity in CHUJANS could clarify risk models and targets for therapy.

## 4. Conclusions

This case underscores the need for comprehensive, syndrome-specific evaluation of sleep disorders in individuals with Chung–Jansen syndrome. To date, published reports have largely relied on caregiver-reported sleep difficulties, highlighting the importance of objective assessment for obstructive sleep apnea and other sleep disorders using polysomnography. The observed feasibility of positive airway pressure therapy in this patient suggests that treatment may be achievable in this rare genetic syndrome, a finding that warrants confirmation in prospective, syndrome-stratified studies. From a sleep medicine perspective, the coexistence of obesity, sleep-related symptoms, and developmental delay or intellectual disability should prompt consideration of PHIP-related disorders, when Prader–Willi syndrome testing is negative. More broadly, early genetic determinants affecting upper-airway anatomy, hypotonia, and particularly obesity—such as PHIP mutations—may manifest clinically as breathing sleep disorders and remain underrecognized in young adults with obesity and neurodevelopmental features. Improved understanding of PHIP-driven disruption of energy homeostasis may further inform targeted therapeutic strategies addressing the molecular basis of genetically driven obesity and its sleep-related consequences.

## Figures and Tables

**Table 1:** Polysomnographic characteristics.

Parameter	Pre-CPAP	On CPAP
Total Sleep Time (min)	310	350
Sleep Efficiency (%)	67.7	81.5
Sleep Stage N1 (%)	15.2	9.1
Sleep Stage N2 (%)	42.2	42.3
Sleep Stage N3 (%)	30.3	21.8
REM Sleep (%)	12.3	26.7
AHI (events/hour)	31.9	0.0
NREM_AHI_	19.8	0.0
REM_AHI_	113.8	0.0
Minimum SpO_2_ (%)	66	89
Average SpO_2_ (%)	89.2	95.4

AHI: Apnea–hypopnea index; CPAP: continuous positive airway pressure; N1: sleep stage 1; N2: sleep stage 2; N3: sleep stage 3; NREM: non-rapid eye movement; REM: rapid eye movement; SpO_2_: oxygen saturation.

## Data Availability

The data presented in this study are available on request from the corresponding author.

## Supplementary Materials

The following supporting information can be downloaded at: https://www.mdpi.com/article/10.3390/ijms27041736/s1.
