# Supplementary material for: Sleep-Disordered Breathing in Chung–Jansen Syndrome
Source: Int J Mol Sci. 2026 Feb 11;27(4):1736. doi: 10.3390/ijms27041736 (PMC12940234; doi:10.3390/ijms27041736)
Supplement: Supplementary file 1 [file ijms-27-01736-s001.zip › ijms-4095160-supplementary.pdf]

Supplementary Table S1. Literature data for Chung–Jansen syndrome (PHIP) cohorts and sleep disturbance.

Study (journal)	Year	N	Age (median; range, years)*	Adults included	Overweight/Obese (%)	Sleep problems (%)	PSG-confirmed OSA reported	Tonsil/Adenoid surgery (%)
Jansen et al., European Journal of Human Genetics [1]	2018	23	14; 5–52	Yes	74.0	18.0	No	NR
Craddock et al., Cold Spring Harbor Molecular Case Studies [3]	2019	10	†9.2; 1.3–15	No (pediatric)	30.0	30.0	No	NR
Kampmeier et al., Frontiers in Cell and Developmental Biology [10]	2023	23	13; 5–54	Yes	69.6	26.1	No	NR
Sudnawa et al., American Journal of Medical Genetics Part A [2]	2024	47	10.9; 0.4–43.7	Yes	55.8	42.6	No	31.9

Percentages are calculated from participants with available data in each study. References correspond to the numbering used in the main manuscript.

Abbreviations: PHIP, pleckstrin homology domain interacting protein; PSG, polysomnography; OSA, obstructive sleep apnoea; NR, not reported.

* Median; range reported where available. † For Craddock et al., the summary age reported is mean 9.2 years (range 1.3–15); median not reported.
